# Interactions Between 2D Materials and Living Matter: A Review on Graphene and Hexagonal Boron Nitride Coatings

**DOI:** 10.3389/fbioe.2021.612669

**Published:** 2021-01-27

**Authors:** João Santos, Matteo Moschetta, João Rodrigues, Pedro Alpuim, Andrea Capasso

**Affiliations:** ^1^International Iberian Nanotechnology Laboratory, Braga, Portugal; ^2^Center for Synaptic Neuroscience and Technology, Istituto Italiano di Tecnologia, Genova, Italy; ^3^Centro de Física das Universidades do Minho e do Porto, Braga, Portugal

**Keywords:** antibacterial properties, two-dimensional materials (2D materials), cellular interaction, neuronal interface, tissue engineering

## Abstract

Two-dimensional material (2DM) coatings exhibit complex and controversial interactions with biological matter, having shown in different contexts to induce bacterial cell death and contribute to mammalian cell growth and proliferation *in vitro* and tissue differentiation *in vivo*. Although several reports indicate that the morphologic and electronic properties of the coating, as well as its surface features (e.g., crystallinity, wettability, and chemistry), play a key role in the biological interaction, these kinds of interactions have not been fully understood yet. In this review, we report and classify the cellular interaction mechanisms observed in graphene and hexagonal boron nitride (hBN) coatings. Graphene and hBN were chosen as study materials to gauge the effect of two atomic-thick coatings with analogous lattice structure yet dissimilar electrical properties upon contact with living matter, allowing to discern among the observed effects and link them to specific material properties. In our analysis, we also considered the influence of crystallinity and surface roughness, detailing the mechanisms of interaction that make specific coatings of these 2DMs either hostile toward bacterial cells or innocuous for mammalian cells. In doing this, we discriminate among the material and surface properties, which are often strictly connected to the 2DM production technique, coating deposition and post-processing method. Building on this knowledge, the selection of 2DM coatings based on their specific characteristics will allow to engineer desired functionalities and devices. Antibacterial coatings to prevent biofouling, biocompatible platforms suitable for biomedical applications (e.g., wound healing, tissue repairing and regeneration, and novel biosensing devices) could be realized in the next future. Overall, a clear understanding on how the 2DM coating’s properties may modulate a specific bacterial or cellular response is crucial for any future innovation in the field.

## Introduction

### Background and Motivation

Other than being present in most of the Earth’s habitats, bacteria live in symbiotic and parasitic relationships with plants and animals. Bacteria are ubiquitous in mammals, and while they are necessary for the development of a balanced immune system, the disruption of microbiomes can negatively affect the health of an individual. Nowadays, bacterial pathogenic agents represent a major public health issue (Medzhitov, 2007; Pope et al., 2008; Ali and Keil, 2009; Ling et al., 2015; Begum et al., 2020; WHO, 2020). The use of antibiotics, one of the main and most effective tools for combating bacterial infections, has gradually led to an increase in drug resistance in the microbes responsible for infections, as a consequence of misuse and/or overuse, requiring ever-increasing doses to achieve the same inhibition effect (Medzhitov, 2007; Pope et al., 2008; Ling et al., 2015; WHO, 2020). Alarming predictions state that at the rate by which bacteria are becoming resistant to antibiotics, drug-resistant bacteria could kill more than ten million people/year by 2050, a prediction that is further aggravated by the increasing ease in global mobility (Medzhitov, 2007; Pope et al., 2008; Ali and Keil, 2009; Ling et al., 2015; Begum et al., 2020; WHO, 2020).

Another concern regarding bacteria is their adhesion to surfaces, and the consequent issue of biofouling. Biofouling refers to the formation of a biofilm consisting of a complex community of one or several microbial species on the surface of an object, which can impair its functionality, e.g., by promoting corrosion. Biofouling and related issues can cause damage to objects, equipment and structures, and often lead to significant financial loss (Flemming and Wingender, 2010). In the context of implanted devices, biofouling should be carefully monitored and prevented to preserve the functionality and lifespan of the implants. Current solutions to minimize biofouling can potentially release toxins and have a harmful impact on the environment and marine life. The realization of effective antibacterial and anti-biofilm coatings based on innovative biocompatible materials would greatly contribute to prevent surface contamination and hindering the spreading of bacteria in many contexts (Szunerits and Boukherroub, 2016).

In medicine, degenerative diseases have overtaken infections as the leading cause of death worldwide, with tissue damage caused by ischemia, stroke and neurological pathologies representing the most prevalent cause of mortality in high-income countries (WHO, 2013). In this scenario, traditional pharmacology shows some limits and challenges (Blaschke, 2009; Enna and Williams, 2009). For these reasons, other strategies (e.g., stem cell therapy and advanced nanomaterials) have been developed with the aim of promoting tissue repair and restoring the correct functionality of the damaged tissue (Howard et al., 2008; Liao et al., 2008; O’Brien, 2011; Wang et al., 2011). Tissue engineering involves replication and regeneration of damaged tissues and organs. To this end, it relies on substrates that mimic the physiological *in vivo* conditions. Suitable substrate materials should be able to guide cell growth and modulation, bioactive molecule delivery, physicochemical cue generation, while having similar mechanical properties to the native tissue (Shin et al., 2016).

Bacteria and mammalian cells have been shown to respond to a wide set of physicochemical signals, such as biochemical, electrical, and magnetic. The interactions with coatings made of different materials have shown to play a role in modulating or tailoring cellular response. Two-dimensional materials (2DMs) are a class of atomic-thick materials that are the subject of intense research efforts, motivated by a wide range of useful properties of high interest in several technological fields (Miró et al., 2014; Bonaccorso et al., 2015; Capasso et al., 2019; Kang et al., 2020). In the biomedical field, applications for 2DMs include bio-sensing (Pumera, 2011), tissue engineering (Goenka et al., 2014), personal protective equipment fabrication (Zhong et al., 2020), and drug or gene delivery (Chimene et al., 2015).

### Graphene and hBN: Structure, Properties, and Role in Bio-Applications

**FIGURE 1:**
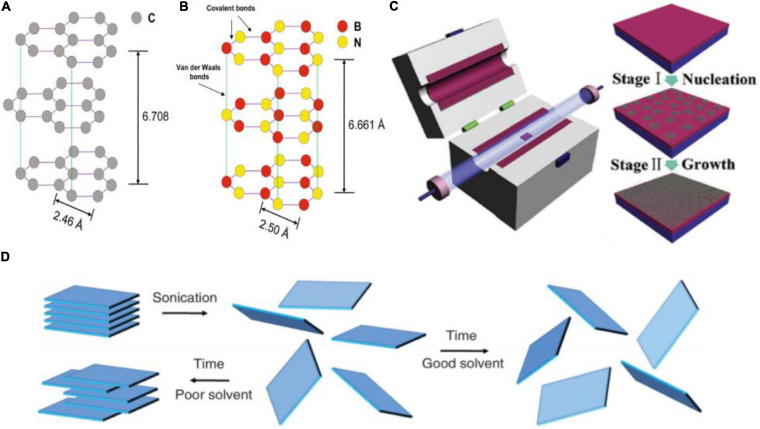
Schematics of graphene **(A)** and hBN **(B)** lattice structure. Reprinted from Kim et al. (2014) Springer Nature. **(C)** General stages of a CVD growth process. Reprinted from Chen et al. (2015) Elvesier. **(D)** Process flow of a top–down approach for the production of exfoliated 2DM flakes in a solvent. The exfoliation of bulk layered materials can occur via ultra-sonication, which provides sufficient energy to overcome the VdW bonds among layers in the bulk crystal and separate them into individual or atomic-thick layers. Reprinted from Nicolosi et al. (2013) Copyright © 2013, American Association for the Advancement of Science.

Graphene and hexagonal boron nitride (hBN) are archetypal 2DMs that have drawn the attention of researchers (Randviir et al., 2014; Kang et al., 2020; Khan et al., 2020; Mendelson et al., 2020). Graphene is a carbon allotrope consisting of a single layer of sp^2^-bonded carbon atoms arranged in a hexagonal lattice (Figure 1A) (Novoselov et al., 2004; Geim et al., 2005; Allen et al., 2010). Monolayer graphene has a thickness of 0.34 nm and a lattice constant of 2.46 Å. When in graphitic, multilayer form, it shows an interlayer distance of 3.354 Å (Kim et al., 2014). Its atomic bonds, combined with its one-atom thickness, grant graphene properties such as high electrical conductivity, thermal conductivity, mechanical flexibility, and optical transparency (Geim and Novoselov, 2007; Lee et al., 2008; Koh et al., 2010). Furthermore, there is the possibility of surface functionalization with a variety of bioactive molecules (Shin et al., 2016). hBN is a layered material with a honeycomb structure analogous to that of graphene, consisting of covalently bonded boron (B) and nitrogen (N) atoms (Figure 1B) (Lynch and Drickamer, 1966). hBN layers have a AA’ stacking configuration bonded by van der Waals (VdW) forces (with interlayer distance of 3.33 Å) (Wang et al., 2017). Having analogous structure but insulating electrical behavior, hBN is sometimes referred to as “white graphene.” hBN features properties such as high hydrophobicity, thermal insulation, electrical insulation, low dielectric constant, resistance to oxidation, high chemical stability, and mechanical strength (Mahvash et al., 2017; Chilkoor et al., 2018; Merlo et al., 2018; Emanet et al., 2019; Mukheem et al., 2019). Due to these properties, 2D BN-based materials have also demonstrated potential in antibacterial coatings (Mukheem et al., 2019), as well as applications in the biomedical field – such as wound healing (Şen et al., 2019), and bone tissue regeneration (Şen et al., 2019; Aki et al., 2020).

Techniques for 2DM production fall within two main categories, defined as bottom–up and top–down (Tour, 2014; Kong et al., 2019; Matsoso et al., 2020). Among the bottom-up techniques, chemical vapor deposition (CVD) allows the production of 2DMs with atomic thickness (i.e., mono- to few-layer) and high crystallinity (Figure 1C) (Lisi et al., 2014; Li et al., 2016; Gnisci et al., 2018; Deng et al., 2019; Faggio et al., 2020). Graphene and hBN are commonly grown on copper (Cu) substrates, which enable a self-limiting growth resulting in monolayer graphene over large areas (Figure 1D) (Faggio et al., 2013; Capasso et al., 2015b; Wu et al., 2015; Deng et al., 2019; Wang L. et al., 2019). After the growth, the 2DMs can be transferred to target substrates via wet etching techniques (Mattevi et al., 2011; Capasso et al., 2014; Backes et al., 2019). CVD is a valid production method especially to fabricate graphene-based devices for electronics and other applications requiring high reproducibility, although it is still a rather expensive technique which requires high temperatures, full control on parameters, and post-production transfer processes (Backes et al., 2019; Deng et al., 2019). The most common top–down technique is liquid phase exfoliation (LPE). In a LPE process, bulk layered materials (such as graphite or hBN crystals) are exfoliated in a liquid solvent using an external source of energy, such as ultra-sonication or shear mixing, as illustrated in Figure 1D. The energy is required to overcome the weak VdW bonds holding the layered bulk crystal together and disperse the exfoliated layers in the solvent. As-produced dispersions of isolated layers (also called “flakes”) can be adjusted in concentration and size-distribution by successive ultracentrifugation stages. The fluidic properties of the dispersions (e.g., surface tension and viscosity) can also be tuned to comply with specific depositions techniques, such as inkjet or screen printing, spray coating, and flexography (Capasso et al., 2015a; Kim et al., 2019). Overall, LPE is a suitable approach to produce liquid dispersions of 2DMs in large amounts, at the expense of a somehow limited control on the thickness and lateral size distribution, and on the defect level of the flakes.

Research on graphene and hBN toward bio-technology has focused to date on three main objectives: (i) new systems capable of inducing tissue regeneration or restoring cell-to-cell communication; (ii) specific diagnostic tools for the *in vivo* detection of biological markers of pathologies; (iii) supports for monitoring and modulating excitable cell activity (Fattahi et al., 2014; Marchesan et al., 2017; Scaini and Ballerini, 2018). Graphene in particular has been proposed to engineer innovative biological interfaces able to adapt and interact with the biological matter (Goenka et al., 2014; Reina et al., 2017). However, the physicochemical interaction between graphene and *in vitro* cell cultures still eludes a full and comprehensive understanding, which will finally shed light on the biocompatibility of these materials (Syama and Mohanan, 2016).

Graphene and hBN have been recently found to induce specific cellular responses in different contexts. Taking into consideration the issues related to bacterial infection and the relevance of degenerative diseases, the different biological interactions of these materials can be exploited toward the following aims: (1) inducing bacterial cell death, e.g., to design medical instruments and mitigate the spreading of infections in hospital environment, and to produce anti-biofouling coatings (Parra et al., 2015c, 2017; Zurob et al., 2019); (2) stimulating cellular growth, such as in the case of tissue engineering, wound healing, and bone tissue regeneration (Fernandes et al., 2019; Şen et al., 2019; Aki et al., 2020). Devices such as epidermal electronics, intra-cortical implants for sensing and brain tissue regeneration require intimate contact with biological systems. Desired properties in these materials are softness, allowing to conform and adhere to the tissue’s micro-roughness, but also biocompatibility (Jeong et al., 2013; Kabiri Ameri et al., 2017; Campos et al., 2019; Meng et al., 2019; Wang Q. et al., 2019); (3) drug delivery and tissue engineering (Edmondson et al., 2014; Goenka et al., 2014; Kumar and Parekh, 2020). As such, the physicochemical features of graphene and hBN, as well as their respective interactions with cells, must be studied. Concerning graphene-based materials (GBM), most of the studies to date focused on the functionalized forms of graphene such as graphene oxide (GO) and reduced graphene oxide (rGO). These materials, however, should be treated as separated cases due to the significant influence of their functional groups and defects in cellular interaction. Regarding hBN-based materials, research is still discontinuous and incomplete. While some forms of BN, such as BN nanotubes (BNNTs) and hBN in liquid dispersion, have been widely investigated in terms of cytotoxicity and possible applications in tissue repairing after injuries (Merlo et al., 2018), 2D hBN coatings for biomedicine are still rather unexplored. Due to the general scarcity of literature regarding hBN, we have decided to extend the review to include more hBN-related materials to provide a more complete outlook.

In this paper, we reviewed the literature describing the various kinds of interactions that pristine graphene and hBN coatings respectively show with different bacterial species and different types of mammalian cells. Following this “Introduction,” the section “2DM coatings and cellular interaction” will report and comment on the main interaction mechanisms taking place when graphene and hBN coatings are used to interface bacterial and mammalian cells. The section “Coating applications” will then build on this by detailing the current and prospective applications for such coatings in the biomedical field. In the “Conclusion,” the main results and perspectives on the topic will be summarized.

## 2DM Coatings and Cellular Interaction

### Graphene and Bacteria

**FIGURE 2:**
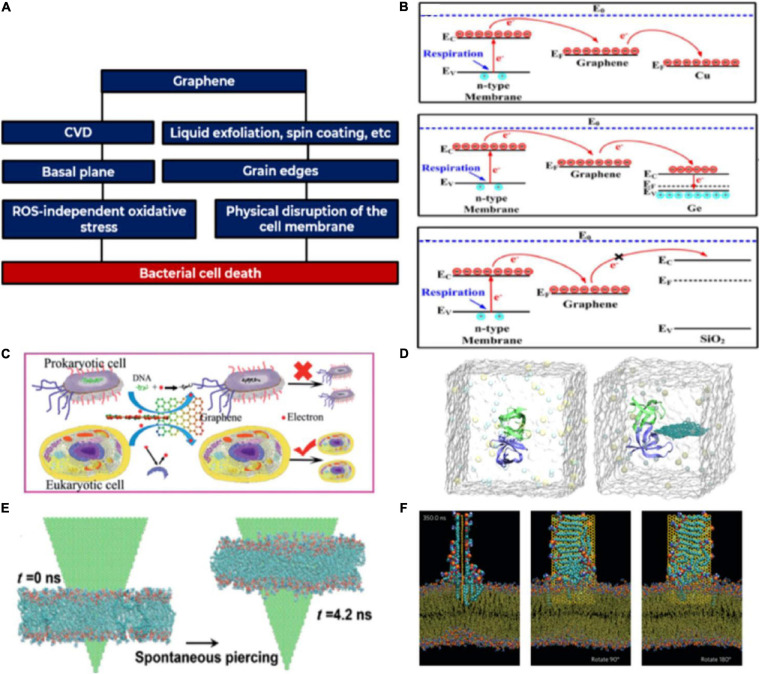
**(A)** General schematic overview of the relationships between different features in graphene coatings. Different production techniques yield graphene coatings with different morphologies, with their respective bacterial interaction mechanisms; **(B)** Influence of the substrate’s electrical conductivity in determining CVD graphene films’ antibacterial activity. Reprinted from Li et al. (2014) Springer Nature; **(C)** Effect of graphene’s electrical conductivity on prokaryotic and eukaryotic cells; the higher complexity of the eukaryotic cell renders it resistant to graphene’s electrical conductivity. Reprinted from Lu et al. (2012), with permission from The Royal Society of Chemistry; **(D)** In the presence of graphene protein denaturation occurs, causing cellular membrane channels to lose their integrity, compromising cell viability. Reprinted with permission from Luan et al. (2015). Copyright 2015 American Chemical Society; **(E)** So-called “nano-knives” effect. Here, there is spontaneous physical penetration of the edge of a graphene flake in the cell membrane causes leakage of cellular material and consequent cell death. Reprinted from Li et al. (2013) National Academy of Sciences; **(F)** Affinity with the lipidic bilayer of the cellular membrane causes phospholipid extraction and consequent loss of cell integrity. Reprinted from Tu et al. (2013) Springer Nature.

In this section, we report on the studied interactions between GBM coatings and bacteria, correlating them to the structural and electrical characteristics of the various coatings. Current literature on the antimicrobial mechanisms in GBMs such as GO and rGO, initially reported in 2010 (Akhavan and Ghaderi, 2010; Hu et al., 2010), has been reviewed by Henriques et al. (2018). The antibacterial activity of graphene-containing composites has also been investigated (Zhao et al., 2016). The antibacterial mechanisms exhibited by graphene and GBMs are commonly found in other 2DMs as well (Mei et al., 2020). Contrarily to most existing works in literature, in this review we focus primarily on pristine graphene coatings. Considering that the physicochemical properties of graphene strongly depend on the production and processing methods (Figure 2A) (Xia et al., 2019), we direct our enquiry to graphene produced by either CVD (Lisi et al., 2017; Kairi et al., 2018; Lin et al., 2018; Deng et al., 2019; Faggio et al., 2020) or LPE (Hernandez et al., 2008; Nicolosi et al., 2013), two common methods for the production of graphene – provided that in the latter case the exfoliated graphene flakes are deposited on a substrate forming a coating. While CVD-deposited graphene is typically atomically smooth (Gnisci et al., 2018; Zhang et al., 2020), coatings made of LPE graphene are often structurally irregular (Nicolosi et al., 2013; Capasso et al., 2015a; Bonaccorso et al., 2016; Niu et al., 2016), since they consist of randomly oriented stacks of flakes (typically deposited by printing or spray coating technique) (Lee et al., 2019).

In the case of atomically smooth, CVD-grown graphene coatings, the main antibacterial mechanism is determined by its electronic conductivity (Figures 2B,C) (Lu et al., 2012; Li et al., 2014). Regarding LPE-graphene, the roughness inherent of these types of coatings has been shown to promote bacterial cell destruction through physical interaction of the cell membrane with the edges of the flakes (Figures 2D–F).

In the case of atomically smooth graphene coatings (such as CVD), where the bacterial cells will not be in contact with exposed flake edges, the antibacterial effects have been shown to arise from electronic conductivity of the basal plane, which induces cell oxidation without reactive oxygen species (ROS) production (Lu et al., 2012; Li et al., 2014). This effect is attributed to the oxidative stress that arises from graphene’s high electrical conductivity, which through disruption of cellular respiration produces oxidative stress and depletion of ATP levels. The antibacterial activity of the basal plane of graphene is mediated through the charge transfer capacity of the underlying substrates, i.e., bacterial cell death occurs only when the graphene film is coupled with an electrically conductive (such as Cu) or a semiconducting substrate (such as Ge). Gram + (G+) *Staphylococcus aureus* and Gram- (G-) (*Escherichia coli*) were used to investigate the antibacterial properties of monolayer graphene film on Cu, semiconductor Ge and insulator SiO_2_. The study showed that while graphene on Cu and Ge could inhibit the proliferation of both bacteria types, the same did not occur on SiO_2_ (Li et al., 2014). On the one hand, G- bacteria have shown more vulnerability in samples with Si substrates than on the ones with SiO_2_ substrates due to negatively charged cellular membrane which enhanced electron extraction. On the other hand, G+ bacteria showed more susceptibility to the physical damage exerted by the vertically aligned graphene due to the composition and shape of its cell wall (Wei et al., 2020). The conclusions regarding the conductivity dependent antibacterial activity, however, are not consensual. Bacterial cells incubated on graphene and hBN (grown by CVD on Cu) did not show any decrease in viability after 24 h, contrarily to cells incubated on bare Cu surfaces. Here, both 2DMs equally acted as physical barriers for the cupric ions, preventing the interaction between bacteria and the Cu substrate, successfully protecting it from biocorrosion (Parra et al., 2015c). CVD-graphene on Cu and Au showed no antibacterial activity. Polycrystalline graphene coatings on Cu, however, allowed for the release of cupric ions due to incomplete substrate coverage, promoting cell death (Dellieu et al., 2015). Proliferation of *E. coli* communities on CVD-grown graphene-on-Au despite the substrate’s electrical conductivity has been reported (Szunerits and Boukherroub, 2016). Usually, graphene’s antibacterial properties stem from ROS-independent oxidative stress. However, there have been reports of graphene-modified commercial water filtering membrane producing an increase in ROS in bacterial cells (Musico et al., 2014).

**FIGURE 3:**
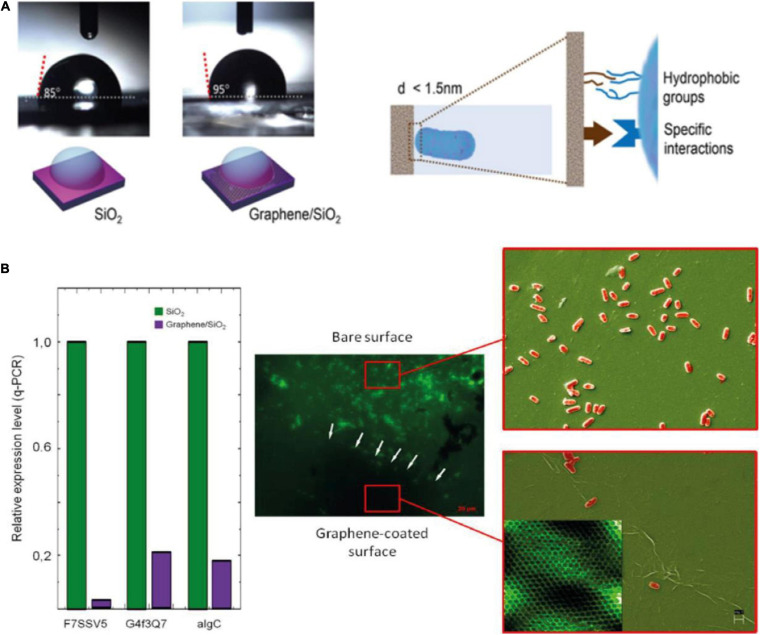
**(A)** Increased hydrophobicity of graphene films prevents bacterial adhesion/wettability-dependent adhesion in graphene coatings. This effect is directly related with **(B)** graphene-influenced lowered relative gene expression levels. Reprinted from Parra et al. (2015a, b) Springer Nature.

Wettability has been reported to play a role in bacterial adhesion. Hydrophilic GO coatings have been compared to hydrophobic rGO and graphene coatings in terms of human plasma proteins and bacterial cells adhesion. Both types of cells showed preferential adhesion to the hydrophobic samples (Henriques et al., 2020). CVD graphene grown on Cu and transferred to SiO_2_ presented a wrinkled surface as a result from the differential thermal expansion on Cu. This situation, coupled with bilayer regions, and micrometric damages resultant from the transfer process, exposed the underlying SiO_2_ and contribute to the overall roughness of the material. They have observed that the coatings impact surface energy and electrostatic interactions with bacteria, decreasing bacterial adhesion through reduction of the expression levels of genes related to adhesion in *Halomonas* spp. CAM2, a typical biofilm producing species. In graphene coated SiO_2_, the substrate has a significant surface state density just below the conduction band, which donates electrons to graphene to balance the chemical potential at the interface. This leads to a n-type graphene coating, repelling negatively charged bacteria in solution, and to an observable decrease in wettability (Parra et al., 2015a) (Figure 3).

Based on these reports, bacterial adhesion appears to be dependent on both the surface texture and electronic properties of the substrate.

Many works have referred to the so-called “nano-knives effect.” This expression usually describes several mechanisms that arise from the contact of the cellular membrane with the edge of a graphene flake: (1) physical insertion of the sharp edges in the membrane and subsequent extraction of phospholipids (Tu et al., 2013; Pham et al., 2015); (2) protein–protein bonding disruption, due to the lipophilic nature of graphene sheets that favorably enter the hydrophobic interface between contacting proteins, leading to their destabilization (Luan et al., 2015). Both of these contribute toward the formation of pores in the membrane and occur usually in LPE-type coatings where the edges of the 2DM are exposed. Pham et al. (2015) have studied the interactions between the surface of LPE multilayer graphene with random orientated edges and *P. aeruginosa* and *S. aureus* cell membranes have been studied through both experimental and simulation methods. Graphene samples with different flake size and orientation were used, resulting in coatings with different surface texture. Their results, shown in Table 1, highlighted the role of roughness of the graphene coating in moderating *P. aeruginosa* and *S. aureus* death. Coatings with penetration angles closer to 90° (with respect to the surface) have more consistently shown to induce the formation of pores in the cell membrane that lead to osmotic imbalance and death. However, the two types of cells attached to the control surfaces remained viable, contrarily to the two other types of fabricated graphene surfaces – rough (GN-R) and smooth (GN-S). The differences in bactericidal activity in the two surfaces can be accounted for by taking into consideration three parameters: exposed edge length (L_GN_), graphene orientation (θ_GN_), and edge length density (*d*_edge_). GN-R exhibits a higher number of viable *S. aureus* cells due to its decreased edge density. *S. aureus* are smaller than *P. aeruginosa*, making it possible for them to colonize the free space between edges (Pham et al., 2015).

**TABLE 1:** Different coating surface parameters and their antibacterial effect against model species (Pham et al., 2015).

Material	L_GN_	θ_GN_	d_edge_	Effective against
GN-R	137.3 nm	62.1°	7.7 μm/μm^2^	*P. aeruginosa*
GN-S	79.7 nm	37.2°	10.8 μm/μm^2^	*P. aeruginosa S. aureus*

This effect is directly related to edge density in the film and the diameter of the bacteria. They have also noted that the number of attached bacteria was variable among the films, with a higher amount being found on smoother surfaces, i.e., pyrolytic graphite surfaces used as control. Pranno et al. (2020) used LPE graphene to improve antimicrobial properties of titanium dental implants, commonly exposed to infections. The graphene flakes were then applied as a coating on the titanium. The lowest % of *S. aureus* biofilm formation was observed in samples with the smaller flake size (obtained with the longest sonication times) (Pranno et al., 2020). Besides bacteria, a physical disruption mechanism has also been recently observed in the case of virus, again via the contact with sharp edges in graphene (Innocenzi and Stagi, 2020). By fully understanding and exploiting these properties, it would be possible to design coatings to protect surfaces from the effects of biofouling; this is particularly relevant for the biomedical field, where implants are typically subject to this kind of degradation.

### Graphene and Mammalian Cells

#### Neuronal Cells

Contrarily to what it has been reported for bacteria, graphene has proven to be suitable for interfacing with mammalian tissues. Despite the existence of a large variety of publications regarding the biocompatibility and the cytotoxicity of GBMs both in *in vitro* (cell lines and primary cultures) and *in vivo* (rodent) models, the majority of them focused on the functionalized forms of graphene: GO and rGO (Pinto et al., 2013; Lalwani et al., 2016). However, the biocompatibility of pristine graphene-coated supports is only partially investigated and usually limited to cell culture models (Li et al., 2011; Park et al., 2011; Tang et al., 2013; He et al., 2016; Veliev et al., 2016; Fischer et al., 2018; Kitko et al., 2018; Pampaloni et al., 2018). Since graphene has been considered as a potential therapeutically tool for neurological diseases treatment, a specific attention has been given to its biocompatibility for neuronal cells (Akhavan, 2016; Bei et al., 2019). Li et al. (2011) fabricated a high-quality CVD graphene coated device and tested its biocompatibility as an interface for primary hippocampal neurons. Authors reported no obvious cytotoxicity, as CVD graphene did not significantly affect cell viability. They speculated that the “friendly” interaction between graphene and neurons is favored by the non-detectability of the catalyst (Cu) contamination traces, usually associated with ROSs formation (Firme and Bandaru, 2010; Li et al., 2011).

**FIGURE 4:**
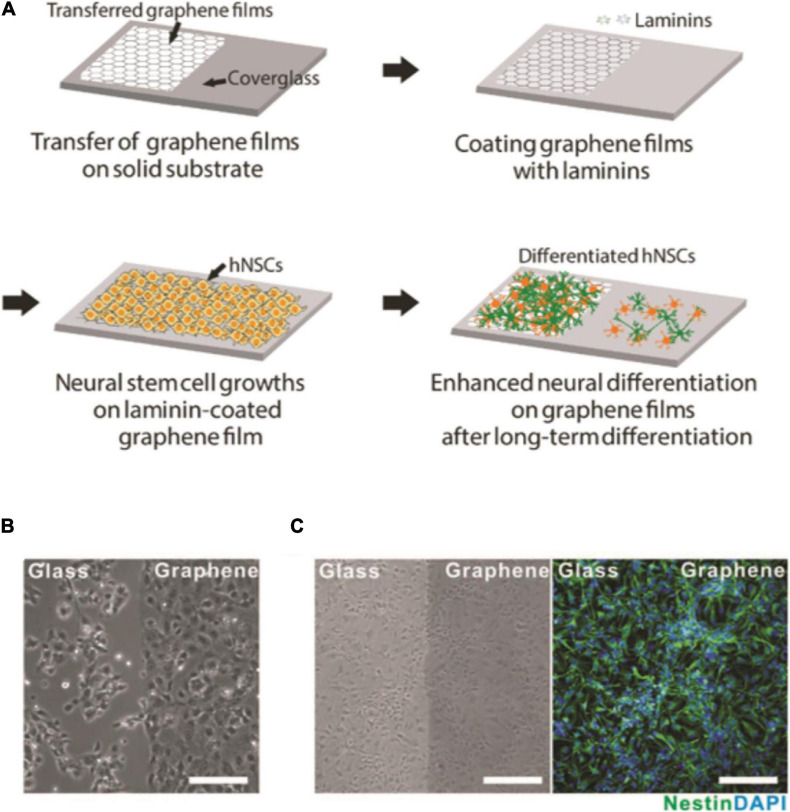
Growth of hNSCs on graphene. **(A)** Schematics of the process. **(B)** Bright-field image of hNSCs on the boundary area between glass (left) and graphene (right) 10 h after cell seeding. Note that more hNSCs were attached on graphene region than on glass region at this early period of cell adhesion. **(C)** Bright-field (left) and fluorescence (right) images of hNSCs proliferated for 5 days. Immunostaining markers were nestin (green) for neural stem cells and DAPI (blue) for nuclei. There was no difference in cell numbers between the graphene and glass regions. Note that all the cells were immunopositive for the nestin marker, indicating they exhibited the property of NSCs. All scale bars correspond to 200 μm. Reprinted from Park et al. (2011) Elsevier ltd.

Veliev et al. (2016) confirmed the observation by Li et al. (2011) while investigating the development of primary hippocampal neurons plated onto monolayer graphene. In according with previous findings, graphene showed a good biocompatibility, but authors attributed that to the high crystalline quality of graphene. It is important to clarify that, in both studies, authors limited the investigation until 5 and 7 days *in vitro* (DIV), respectively (Li et al., 2011; Veliev et al., 2016). More recently, Pampaloni et al. (2018) tested the interaction between monolayer graphene and neurons, again restricting the biocompatibility evaluation to 8–10 DIV. In accordance to previous studies, authors confirmed the excellent biocompatibility of the material, reporting no differences in cell viability. Due to the presence of astrocytes in the primary cultures, authors also evaluated the influence of graphene on astrocytes, indicating no evident cytotoxic effect on glial cells and no changes in neurons/astrocytes ratio (Pampaloni et al., 2018). He et al. (2016) extended the time of cytotoxicity investigation, monitoring neuronal cultures for 21 days: They did not found any evident cytotoxicity and confirmed the excellent biocompatibility of graphene–coated substrates. Indeed, 4 h after the plating, neurons were correctly adhered and spread out compared to ones grown in control conditions. They ascribed the good biocompatibility to the substrate surface morphological characteristics. Starting from the assumption that material surface structure has an impact on cellular adhesion (Li et al., 2010), He et al. (2016) suggested that the presence of ripples and wrinkles might result in an increased mechanical interlocking between graphene and hippocampal neurons. Interestingly, Kitko et al. (2018) ascribed to cholesterol the good neuron adhesion into graphene. Previously, Zhang et al. (2016) had demonstrated through computational modeling that graphene interacts preferentially with cholesterol (specifically localized at the level of the eukaryotic membranes), causing its extraction (Zhang and Wang, 2015). Starting from this assumption, Kitko et al. (2018) confirmed experimentally the computational prediction, demonstrating that graphene is capable to increase cell membrane cholesterol concentration. Authors did not limit their study to neurons, but replicated the experiments taking in consideration fibroblasts. Again, they showed an analogous cholesterol increase in fibroblasts grown onto graphene, concluding that cholesterol should be considered as a graphene effect mediator (Kitko et al., 2018). Specific attention has also been given to human neuronal stem cells (hNSC). Park et al. investigated the effect of graphene-coated solid substrates on hNSCs. Authors did not report any harmful effect of graphene on the progenitor cells that maintained their biochemical properties (positivity to nestin immunostaining), confirming the good biocompatibility of the support (Figure 4) (Park et al., 2011). Same results have been confirmed by Tang et al. (2013). However, in this work, authors grew hNSCs onto graphene nanospheres. They reported that cells tightly adhered to the substrates without any evident cytotoxic response (Tang et al., 2013). Similarly, a graphene-based foam was shown to act as biocompatible scaffold for the culture of human neurons, supporting cell viability and differentiation of human embryonic stem cell-derived cortical neurons (D’Abaco et al., 2018).

A controversial aspect, which is necessary to mention, concerns the common practice of pre-coating graphene-based substrates. The practice of pre-coating graphene with poly-L-lysine (PLL) before neuronal seeding has been used by Li et al. (2011) and later by Veliev et al. (2016). Due to its high hydrophilicity, PLL is known to be an important factor for correct cell adhesion to non-biological substrates, by mimicking the extracellular matrix. Authors suggested that a PLL coating might be an important factor that has favored neuronal adhesion and promoted cell viability. Indeed, neurons grown onto PLL-coated graphene exhibited a larger cell body size and a highly developed dendritic arborization with long and branched neurites (Li et al., 2011; Veliev et al., 2016). Alternately, both Park et al. (2011) and Tang et al. (2013) treated graphene and control glass with laminin to facilitate hNSC adhesion. In addition, He et al. (2016) clearly stated the impossibility of growing neurons onto graphene without coating. Authors excluded the interference of PLL-coating on the presented data and suggested that the good cell adhesion on the substrate is related exclusively to the graphene surface properties (He et al., 2016). Nonetheless, at the same time, Veliev et al. demonstrated the possibility to grow neurons onto bare graphene. In accordance with Veliev et al. and Kitko et al. (2018) managed to properly seed neurons on bare CVD graphene, without the need of a pre-coating treatment. However, authors underlined that the coating absence does not permit a total interaction between neurons and the substrate. Indeed, this approach did not prevent the deposition of biomolecules (present in the serum containing media) that could form a protein corona (previously demonstrated for GO, Hu et al., 2011). Pampaloni et al. (2018) confirmed the possibility to grow neurons onto graphene without any pre-coating step. But, it is necessary to report that authors used non-pure neuronal cultures (Pampaloni et al., 2018). The presence of glial cells must be seriously considered, since astrocytes are known to favor neuronal adhesion, neurite elongation and synapse formation and maturation (Laming et al., 2000; Chung et al., 2015). Because of the variety of discordant studies, PLL use to promote cell adhesion remains a controversial aspect in the study of the interaction between graphene and mammalian cells. The mechanism by which neuronal cells properly adheres to graphene without a pre-coating treatment is still unclear and needs to be further investigated. Indeed, it becomes necessary to deeply evaluate the chemical interaction between PLL and graphene, to avoid data misinterpretation.

#### Non-neuronal Cells

Beyond the possible application in neurological diseases/disorders, graphene also represents an attractive material for non-neuronal tissue engineering. The possibility to use graphene as a platform that supports and promotes cell adhesion, proliferation, and maturation, pushed the researchers to investigate its interaction with non-excitable cells. Kalbacova et al. (2010) tested the capability of human non-neuronal cells to grow onto monolayer and multilayer graphene. Authors plated three distinct types of cells: human osteoblasts, SAOS-2 cells (a cell line) and human mesenchymal stromal cells (MSCs, primary cells). 48 h after the plating, both osteoblasts and MSCs homogenously covered all the graphene substrate, verifying the biocompatibility of the substrate (Kalbacova et al., 2010). Subsequently, three independent studies reached the same conclusions as Kalbacova et al. (2010). Nayak et al. (2011) verified the biocompatibility of graphene for mesenchymal stromal cells (MSCs), reporting no changes in cell viability between MSCs plated onto graphene-coated and non-coated supports and concluding that cell seeding was not negatively affected by the graphene presence. Aryaei et al. (2014) confirmed the biocompatibility of the graphene supports for osteoblast cells, investigating viability and adhesion. Authors reported no toxic effects of graphene across all substrates independently by the different surface properties (i.e., thickness and roughness) (Aryaei et al., 2014). Park et al. further investigated the CVD-graphene interaction with the mesenchymal stem cells, focusing on the possibility to promote the stem cell differentiation into cardiomyocytes. Firstly, authors displayed that graphene did not alter cell viability. Indeed, no significant changes were showed in terms of living cells between MSCs plated onto graphene-coated or uncoated supports, at different time points. In addition, cells plated onto graphene presented an increased expression of Bcl-2 (anti-apoptotic marker) and a reduced expression of Caspase-3 (pro-apoptotic marker), but the degree of cell proliferation was similar across the conditions (PCNA expression) (Park et al., 2014). In more recent years, Rodriguez et al. (2017) and Xie et al. (2017) studied the biocompatibility of the graphene film for human dental pulp stem cells (DPSC). In both works, authors demonstrated that DPSC were able to adhere correctly to graphene coated substrate as early as 1 day after the seeding and properly proliferate, despite material high hydrophobicity (Rodriguez et al., 2017; Xie et al., 2017).

Aiming at an extended knowledge about the interaction of graphene with mammalian cells, it is also useful to study how graphene can interact with cancer cells. In a way similar to its interaction with bacteria, graphene shows toxic effects when put in contact with tumor derived cells. Despite the need for further investigation in this field, in recent years some preliminary studies suggest the possibility to use graphene as anti-neoplastic agent. Zhou et al. (2014) studied the interaction between graphene in a liquid solution and human breast cancer cells (MDA-MB-231). Authors demonstrated that graphene leaded to the inhibition of electron transfer chains, a consequently reduction in ATP production and impairment of F-actin cytoskeleton assembly, crucial mechanisms to promote migration and invasion of metastatic cells (Zhou et al., 2014). In addition, both GO and B-rGO, also in liquid solution, have shown toxicity toward MCF-7 human breast cancer cells in a dose-dependent manner, as shown by a decreased cell viability, associated with an increased ROS production and release of lactate dehydrogenase (Gurunathan et al., 2013). However, studies regarding the interaction of graphene with virus and cancer cells are still limited, and further research will be thus needed in the future. Monolayer graphene grown by CVD was studied as a coating for porous Ti substrates for bone repair. The coatings revealed to be biocompatible and to favor cellular growth and adhesion, thus improving the properties of the substrate. The positive results were attributed to the impermeability of graphene to metallic ions, preventing the contact between cells and metal ions in the porous structure (Lascano et al., 2020).

**FIGURE 5:**
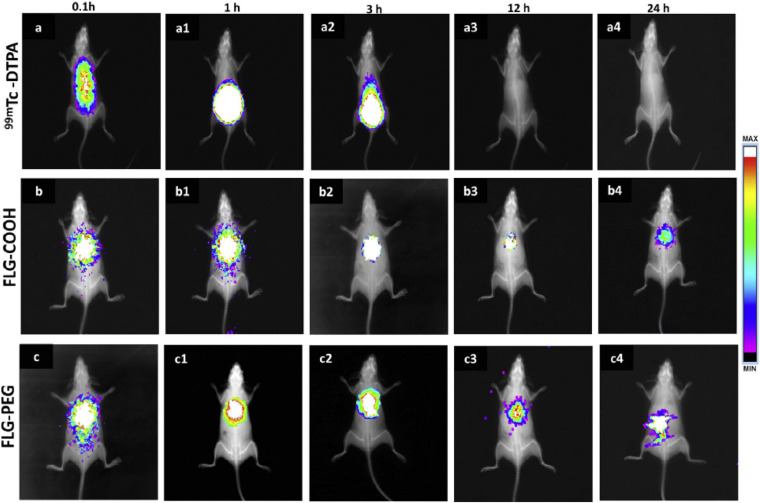
Real-time *in vivo* organ biodistribution analysis using Technetium-99 m (radionucleide) labeled graphene in mice. The signal was accrued for 24 following *i.v.* injection of **(a–a4)** ^99m^Tc-DTPA alone **(b–b4)** ^99m^Tc-FLG-COOH and **(c–c4)** ^99m^Tc-FLG-PEG. Reprinted from Sasidharan et al. (2015) Elsevier ltd.

The number of *in vivo* studies investigating the toxicology, distribution and clearance of graphene is gradually increasing. Nonetheless, the vast majority concerns the functionalized form of graphene, in particular GO, given its capability to better interact with the biological matter (Nurunnabi et al., 2015; Lalwani et al., 2016; Lu et al., 2019). For these reasons we extended our attention by including graphene nanosheets and nanoplatelets (GNP). Yang et al. (2011) studied for the first time the long-term *in vivo* distribution and toxicity of 125I-labeled graphene nanosheets functionalized with polyethylene glycol (PEG). Authors treated intravenously mice with graphene nanosheets at 20 mg/kg for 3 months and demonstrated that the material localized preferentially at the level of the reticuloendothelial system (RES), liver and spleen. Interestingly, graphene nanosheets were gradually cleared by both renal and fecal excretion. No significant toxicity was detected by blood analysis and histological examinations, leading authors to encourage further studies in this field (Yang et al., 2011). Between 2011 and 2013, three independent works investigating the *in vivo* toxicity of graphene at the level of the lung were published (Duch et al., 2011; Schinwald et al., 2012; Ma-Hock et al., 2013). Duch et al. (2011) studied the *in vivo* toxicity of three distinct GBMs: solution of aggregated graphene, dispersed graphene into pluronic acid and GO and administered them intra-tracheally into the lung of young mice. Interestingly, severe tissue injuries were reported in mice treated with GO persisting until 21 days after the administration; on the contrary pristine graphene showed a significant reduction in toxicity. Authors demonstrated that GO induces a covalent oxidation and boosts pulmonary toxicity by enhancing mitochondrial ROS species formation, tissue inflammation and cell apoptosis, concluding that the 2D-graphene is a safer option for a biomedical application (Duch et al., 2011). Schinwald et al. (2012) used commercially available GNPs (consisting in few layers of graphene) and evaluated their breathability, deposition and eventual inflammatory potential. They demonstrate that GNPs (up to 25 μm of diameter) were respirable and deposited beyond the ciliated airways. Interestingly the GNPs revealed to be inflammogenic in both lung and pleural space. Indeed, several inflammatory markers were found after both the bronchoalveolar lavage, the pleural lavage and the *in vitro* assay. Authors stated that GNPs are a potential risk for the human health and the reduction of their diameter is needed for biomedical applications, given the capability of macrophages to phagocyte them (Schinwald et al., 2012). Ma-Hock et al. (2013) exposed male Wistar rats for 6 h per day on 5 consecutive days and evaluated the toxicity after the end and after 3 weeks from the exposure. They reported an increase in the inflammatory processes at exposure concentration of 10 mg/m^3^ (Ma-Hock et al., 2013). The authors of the three works agreed in suggesting a deeper investigation of the *in vivo* cytotoxic of the graphene before any human application. Sasidharan et al. (2015) presented a 3-month report that studied the acute and chronic toxicity of intravenously administered graphene in male Swiss albino mice. For their work, authors choose to administer 20 mg/kg of few layer graphene (FLG) and its derivates carboxylated FLG and PEGylated FLG. They demonstrated that during the first 24 h graphene accumulated preferentially in the lung, which represented the tissue with the highest uptake and retention. In addition, graphene was found also in spleen, liver, and kidney, but no accumulation was detected in brain, heart, or testis (Figure 5). FLG caused severe cellular and tissue damages. Necrosis, fibrosis, hepatic and renal injuries and glomerular dysfunction were detected in the organs where graphene was accumulated. Molecular analysis revealed that 23 markers of critical inflammation and immune response were altered in gene expression. On the contrary, FLG-PEG induced no significant toxicity, despite its persistence in liver and spleen after 3 months. Authors concluded that graphene functionalization is the safer root for the biomedical application of this material (Sasidharan et al., 2015).

In the same year, Shin et al. (2015) published a promising work investigating the graphene *in vivo* toxicity by nasal inhalation system Sprague-Dawley rats. Animals were treated for 6 h/day for 5 days and evaluated the recovery for 1, 3, 7, or 28 days. Authors took in consideration three distinct groups: (i) rats treated with control ambient air; (ii) rats treated with a low concentration of graphene (0.68 mg/m^3^); and (iii) rats treated with high concentration of graphene (3.86 mg/m^3^). No significant alterations were detected in animal and organ weight and in the levels of protein expression (i.e., lactate dehydrogenase and albumin). Despite alveolar macrophage ingestion of graphene was observed in both material-treated groups, no severe toxic effect was detected at the concentration and time points used (Shin et al., 2015). Mao et al. (2016) studied the long-term *in vivo* distribution of graphene in mice after inhalation associated with precise graphene quantification. Authors treated mice with a carbon-14 labeled FLG by oral gavage or intratracheal instillation and quantify the distribution of FLG for up to 3 or 28 days. Mice treated intratracheally showed a FLG retention in the lung of about 47% with a dose-dependent acute lung injury and pulmonary edema. Interestingly the toxicity resulted reduced with time despite the continued presence of FLG in the lung. 1 and 0.18% of FLG was detected in liver and spleen, respectively after 14 days and in feces after 28 days. No gastrointestinal absorption was detected in animals treated with FLG by oral gavage. In this robust work, authors demonstrated the partial persistence of graphene in the lung (causing only transient cytotoxicity) and, in accordance with Yang et al. (2011), its capability to be eliminate by feces (Mao et al., 2016). In Jia et al. (2019) highlighted the *in vitro* (HEK cells) and *in vivo* (zebrafish) toxicity of graphene and GO of three different sizes (small, medium, and large). Authors demonstrated that *in vitro* both the small and the large size of graphene and GO increased DNA damage, ROS formation and the expression of associated critical genetic markers. Injection of both graphene and GO in zebrafish induced ROS generation and developmental alterations. In general, a significant higher toxicity was reported for smaller size graphene (in particular for GO) that showed a stronger ability to decrease the survival rate and induce the acute toxicity (Jia et al., 2019).

### hBN and Bacteria

**FIGURE 6:**
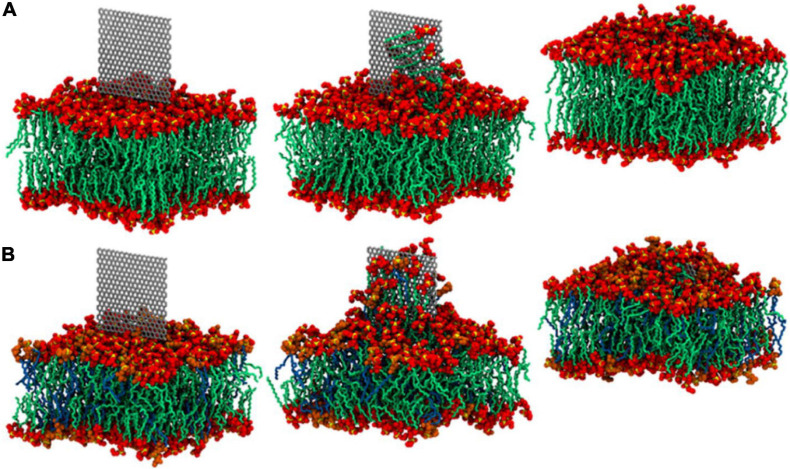
hBN flakes cause lipid extraction, as shown through MD simulation. **(A,B)** Represent the outer and inner membranes present in a G- organism, respectively, after 0, 50, and 500 ns. The continuous extraction of phospholipids from the membrane’s bilayer causes damage and subsequent loss of integrity. Reprinted with permission from Zhang et al. (2019). Copyright 2019, American Chemical Society.

Compared to graphene, the current literature focusing on the interactions between hBN-based materials with cells is rather limited. For this reason, in addition to hBN-based coatings, in this paragraph we report also on the main studies conducted on hBN materials and nanocomposites dispersed in liquids (i.e., not deposited as coatings in solid state), including other BN structures. Kıvanç et al. (2018) investigated the effect of hBN NPs in *S. mutans* 3.3, *S. pasteuri* M3, C*andida* sp.*M25*, and *S. mutans* ATTC 25175 specimens. The used concentration of hBN NPs did not result in bacterial cell death but inhibited bacterial biofilm growth (Kıvanç et al., 2018). Studies conducted using hBN nanoflakes produced by LPE suggest that they can physically damage bacteria cells by disrupting their membrane and consequentially releasing intracellular material. Zhang et al. (2019), via experiments and molecular dynamics (MD) simulations, revealed that phospholipids are attracted to the surface of BN, leading to the nanosheet insertion and destructive lipid extraction, with the hydrophobic effect having a role in the extraction process. In their work, they modeled the inner and outer membranes of G- specimens and uncovered a phospholipid extraction effect in the presence of hBN flakes, akin to the extraction effect exhibited by graphene (Figure 6) (Zhang et al., 2019).

Similarly, Li Z. et al. (2018) simulated the lipid extraction caused by single BN nanosheets and reported a correlation between changes in the lipid extraction behavior and temperature, due to the lipid membrane phase transition. Pandit et al. (2019) demonstrated that oriented BN flakes in a low-density polyethylene matrix can have bactericidal effects when the extruded nanocomposite is in contact with several types of bacteria. The findings demonstrate that the sharp-edged BN nanoflakes changed the cells envelope morphology due to substantial physical damage, leading to lysis of the bacterial cells (Pandit et al., 2019). Antimicrobial composites were produced by grafting quaternary ammonium compounds to the surface of hBN nanoplatelets and using the modified nanoplatelets as filler for linear low-density polyethylene. The nanocomposite was 100% effective in inhibiting *E. coli* and *S. aureus* bacterial growth. The excellent antimicrobial activity is attributed to direct contact mechanisms, which allows to avoid the use of biocides and consequently reduce environmental pollution (Xiong et al., 2019). Gudz et al. (2020) showed that both pristine BN films and gentamicin- and amphotericin-loaded films successfully inhibited the growth of antibiotic resistant G- *E. coli* K-261. The immersion of the BN film in normal saline solution generates ROS species, which can lead to accelerated oxidative stress at the site of physical cell damage (Gudz et al., 2020). The antibacterial activity of a polyhydroxyalkanoate, chitosan, and hBN-incorporated nanocomposite was investigated through a time-kill method against multi drug resistant bacteria, such as methicillin-resistant *S. aureus* and *E. coli* (K1 strains) bacteria. The results showed significant antibacterial activity (Mukheem et al., 2019). BN nanosheets produced by chemical exfoliation and subsequently doped with varying concentrations of Cu showed excellent catalytic activity for dye degradation and treatment of industrial wastewater. The Cu-doped BN nanosheets also showed potential as antimicrobial agents against *S. aureus* and *E. coli* bacteria (Ikram et al., 2020).

The current state of the art on the mechanisms of interaction of hBN with bacteria is still in nascent stage. Further research is needed to clarify the mechanisms, possibly in comparative terms to graphene, whose high electrical conductivity seems to play an important role in mediating its interaction with cells.

### hBN and Mammalian Cells

In the last decade, studies on the interaction between mammalian cells and BN nanotubes (BNNTs) have dominated the vast majority of publications, leaving contrasting results, and unanswered questions (Chen et al., 2009; Ciofani et al., 2010a, b, 2014; Lahiri et al., 2010, 2011; Horváth et al., 2011; Jiang et al., 2015). In recent years, the soluble forms of hBNs, i.e., nanoparticles (NPs) and nanosheets, have been considered (Kıvanç et al., 2018; Mateti et al., 2018; Taskin et al., 2020), while the toxicity of 2D hBN films and possible cell interfacing applications were not investigated. Reviews regarding the applications of different BN structures have pointed out important works regarding the interactions with mammalian cells and their cytotoxicity (Pan et al., 2020). hBN and BNNTs in general have been considered as good candidates for a wide range of applications in the biomedicine (e.g., drug and gene delivery, tissue-mimicking biomaterials), pharmaceutics and cosmetics (Emanet et al., 2019). For these reasons, similarly to the chapter before, we extended the literature survey to hBN materials in solution.

**FIGURE 7:**
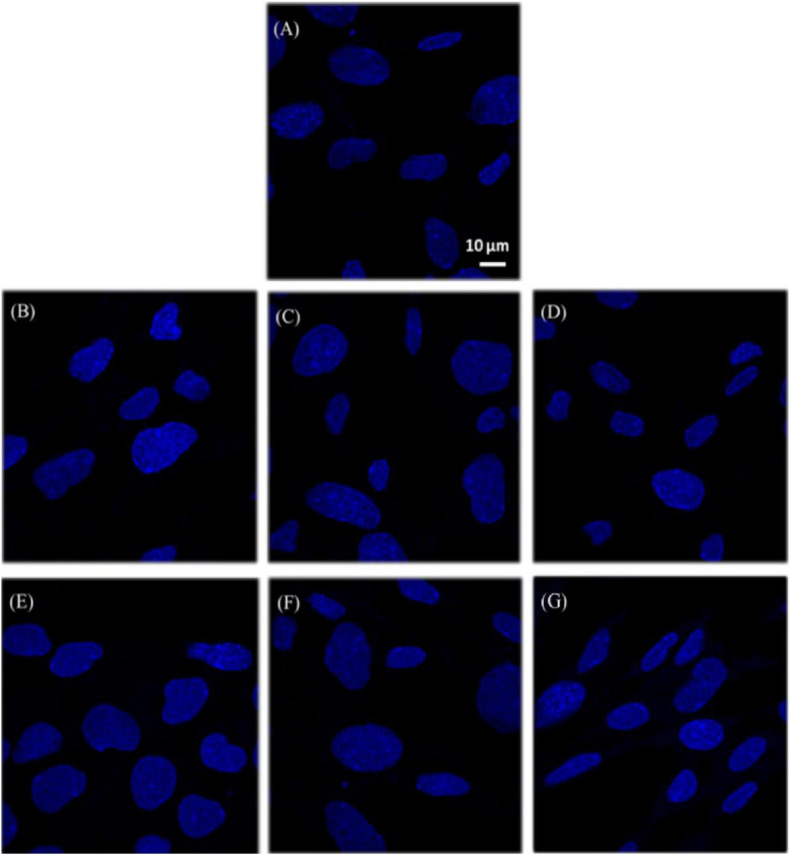
Detection of apoptotic bodies on embryonic mouse hippocampal (mHippoE-14) cells treated with hBNs and BA exposed for 72 h. **(A)** Control cells, **(B)** 4.4, **(C)** 22, and **(D)** 44 μg/mL B containing hBNs with 72 h exposure, and **(E)** 4.4, **(F)** 22, and **(G)** 44 μg/mL B containing BA with 72 h exposure. Reprinted from Irem et al. (2019).

Mateti et al. (2018) studied the biocompatibility of hBN nanosheets and NPs of different dimensions for osteoblast-like cells (SaOS2). They obtained NPs with a diameter range between 100 and 200 nm and two distinct groups of nanosheets (NS1, diameter: 1 μm and thickness: 100 nm; NS2, diameter: 100 nm and thickness: 3 nm). Authors tested the cytotoxicity of the materials for SaOS2 cells and reported a significant decrease in cell viability in presence of both nanosheets and NPs with the smallest size. They attributed the cytotoxicity of such small NPs to their capability to be internalized by the cell, triggering ROS formation (Mateti et al., 2018). Taskin et al. (2020) studied and tested the biocompatibility of hBN flakes *in vitro* on a mouse hippocampal cell line (mHippo E14). Authors treated cells with hBN and its degradation product (BA), fixing the range of exposure at 4.4–440 μm/mL (for 24 and 72 h). No cytotoxic effects, no changes in cell cycle, ROS production and DNA damage were detected for hBN concentration lower than 22 μm/mL. In addition, both hBN and BA favored the cell survival after exposure to doxorubicin (an anti-neoplastic agent), by reducing oxidative stress (Figure 7) (Taskin et al., 2020).

Kıvanç et al. (2018) investigated the effect of hBN NPs in two mammalian cell lines: human skin fibroblasts (CCD-1094Sk, ATCC^®^ CRL 2120^TM^) and Madin-Darby canine kidney cells. At the highest concentration of 0.4 mg/mL, the hBN NPs caused mild cytotoxic effect on CRL-2120 cells (Kıvanç et al., 2018). Polyvinyl alcohol/hBN/bacterial cellulose (BC), 3D-printed composites have been used as bone tissue scaffolds. Significant increase in human osteoblast cell viability on the scaffolds was observed for composites with specific weight ratios. The work deduced that BC-doped, 3D-printed scaffolds with well-defined porous structures have considerable potential in bone tissue engineering. hBN contributes to improve the mechanical properties of the composite, as well as the thermal stability and swelling degree (Aki et al., 2020). Chemically functionalized BNNTs have shown biocompatibility in *in vitro* assays on fibroblast cells (Ciofani et al., 2012). BNNTs have been investigated as boron atom carriers in boron neutron capture therapy, a treatment for several forms of aggressive cancer, including cerebral glioblastoma multiform. *In vitro* results have pointed toward selective uptake of these nanotube vectors by glioblastoma multiforme cells, but not by normal human fibroblast (Ciofani et al., 2009).

hBN synthesized from BA has been used as a therapeutic route in wound healing, due to their biodegradability and ease of dispersion in aqueous environment. Cellular uptake capacities were determined, and human umbilical vein endothelial cells (HUVEC) had higher uptake capacity when compared to human dermal fibroblasts, explaining the different proliferation effect of hBN in these cell lines. Nevertheless, improvement of proliferation and migration in both types of cells was observed. Angiogenesis ability of HUVECs treated with hBN showed promising results. Additionally, it was found that hBN might also improve the wound healing process by lowering ROS due to its antioxidant capacity, similarly to BA; it was found that hBN can rescue cells from apoptosis, and that at low concentrations, it did not depolarize the mitochondria nor disrupt F-actin formation (Şen et al., 2019).

The dose-dependent effect of hBN NPs on biological systems was investigated *in vivo* in Wistar albino rat subjects. Biochemical, hematologic and histopathology parameters were examined for 24 h after intravenous injection of different doses of hBN NPs. Hematological and biochemical parameters showed no changes except in the 1600 and 3200 μg/kg dose groups. Histological detections on these groups indicated the hBN NP treatment induced significant damage in the liver, kidney, heart, spleen, and pancreas. The results also indicated that hBN NPs with diameter of 121 nm could hold promise in biomedical applications, where low doses between 50 and 800 μg/kg are not toxic (Kar et al., 2020). Parkinson’s disease (PD) is an aggressive neurodegenerative disease characterized by the loss of dopamine-sensitive neurons in the substantia nigra region of the brain. hBNs demonstrated neuroprotective properties in the experimental PD model induced by 1-methyl-4-phenylpyridinium (MPP^+^). Cell viability tests confirmed that hBNs do not exhibit neurotoxic effects. Flow cytometry analysis determined that hBN significantly decreased apoptotic cells in the experimental PD model (Küçükdoğru et al., 2020). Overall, although less studied than GBMs, hBN, and hBN-based materials have shown promise in a wide range of biomedical applications.

## Coating Applications

As detailed in Section “2DM coatings and cellular interaction,” graphene and hBN present complex interaction in biological medium mediated either by the direct edge contact and membrane disruption, or through electrical conductivity. In the case of bacterial cells, the features of coatings made from these materials have resulted in decreased adhesion and cell destruction. However, in the case of mammalian cells, not only are these mechanisms innocuous, but they are advantageous in promoting tissue growth and cell differentiation through enhanced adhesion. Exploiting these features allow the design of coatings for different ends, with cell-specific tailored interactions. In the following section we show how these materials have a positive impact in preventing biofouling by decreasing cellular adhesion, and how they can be used to enhance mammalian cell growth.

### Graphene and hBN for Antifouling

Corrosion accounts for a significant economic cost annually, thus motivating numerous efforts to prevent it (Gerhardus and Brongers, 2010; DNV GL, 2015). One type of corrosion comes from biofouling, which encompasses the formation of a microbial biofilm on a metallic surface. Microorganisms growing on surfaces perform a variety of metabolic reactions, with products that promote the deterioration of the underlying substratum, ultimately leading to the mechanical failure of the surface they are attached to. The biofilm contains exopolymers which impede the diffusion of solutes and gases between the surface and the bulk aqueous phase. These exopolymeric substances are essential for the biofilm since they allow the development of highly structured microbial communities on the surface by enhancing adhesion to the metallic surface and providing stability to the biofilm. The various species can collectively carry out metabolic activities that are potentially more corrosive to the underlying surface than could be achieved by a single species alone. These features of sessile microbial growth represent important prerequisites of biocorrosion. Additionally, bacterial species within the biofilm are more resistant to antibiotic and UV radiation that those in their planktonic or sessile state, thus requiring novel approaches in order to mitigate (Otter et al., 2015). Currently, the techniques used to control biofouling include physical (heat treatments, pulse power-technology, radioactive coatings, flushing, scrubbing and biological control) and chemical methods (biocides, chlorine, marine bioactive compounds, Ag, or Cu alloys). However, these approaches are non-specific, which leads to environmental impact resulting from their effect on species not involved in biofilm formation (Parra et al., 2015a). While typical approaches rely on the application of coatings with bactericidal payloads, these present limitations such as low durability and gradually lead to bacterial resistance development as well as negative environmental impacts (Reed et al., 2019; Zurob et al., 2019). Other control strategies imply the use of polymeric coatings, such as epoxy. This type of coating acts as a barrier against water, oxygen, and corrosive species. Despite their advantageous properties, such as low toxicity, these are a temporary solution, due to their high brittleness, poor impact resistance and flexibility, leading to microscopic cracks and mechanical damage (Parra et al., 2015b; Chhetri et al., 2019).

**FIGURE 8:**
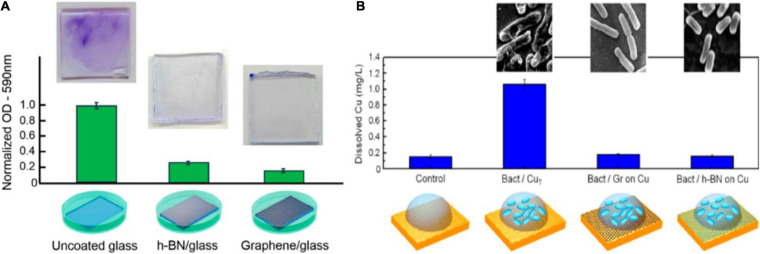
**(A)** Optical density (OD) and crystal violet results for *E. cloacae* biofilms grown on uncoated glass, h-BN-coated glass, and single layer graphene (SLG)-coated glass samples. Reprinted from Zurob et al. (2019). **(B)** Atomic absorption spectroscopy (AAS) measurements on coated and uncoated Cu samples after 24 h of bacteria contact showing Cu dissolution. Dissolved Cu for h-BN- and graphene-coated Cu samples in contact to bacteria is below the detection limit, as well as control sample. Only bare Cu_T_ in contact with bacteria present Cu dissolution. Reprinted with permission from Parra et al. (2015c). Copyright 2015 American Chemical Society.

Here, we contemplate the interaction mechanisms of graphene or hBN with bacterial cells as the basis for effective antibiofouling coatings. These materials have demonstrated superior functionality in comparison to the other above-mentioned approaches due to their rich biological interaction mechanism, explored in the prior sections, which present the development of more resistant bacteria. Graphene and hBN coatings have shown to be ion impermeable barriers due to their reduced lattice size, which is smaller than the bacteria and their metabolites thus creating a physical barrier that prevents the bacteria from interacting, and consequently corroding the underlying substrate. Parra et al. (2015c, 2017) have shown that this mechanism is effective in controlling MIC in Cu and Ni substrates. Zurob et al. (2019) studied the effect of graphene and hBN coatings on a wild Gram- *Enterobacter cloacae* strain biofilm. Graphene-coated glass exhibits 83.6% less biofilm than uncoated glass. In the case of hBN, a 73.8% suppression of biofilm formation was found (Figure 8). While both coatings contribute to a significant reduction of the biofilm, no bactericidal effects were found, suggesting that the bactericidal effect is independent of the charge transfer capabilities of the substrate, and that biofilm reduction could be attributed to decreased cellular adhesion. The authors mention the influence of electrostatic interactions and surface energy rather than charge transfer in antibacterial properties (Zurob et al., 2019). The performance of CVD hBN and graphene coatings in preventing cellular adhesion are shown in Figure 8. Parra and Zurob agreed that bacterial adhesion inhibition is dependent on surface energy and electrostatic interaction. Simultaneously to the ability to combat microbial corrosion, hBN coatings can be also effective at suppressing galvanic effects due to their insulating nature, unlike graphene, where the local defects act as a cathodic site for anchoring and reducing terminal electron acceptors, enhancing the negative effects of galvanic corrosion. This was demonstrated by Chilkoor et al. (2018) where a CVD-grown monolayer hBN was used to protect a Cu substrate from planktonic and sessile forms of *D. alaskensis G20*, a sulfate-reducing bacterium. The monolayer hBN acted as an impermeable layer for the corrosion effects of the biofilm, blocking the migration of aggressive metabolites to the substrate and subsequent degradation (Chilkoor et al., 2018).

Al-Saadi et al. (2017) reported the beneficial impact from the impregnation of hBN into a silane composite for the purpose of bioimplant coatings, successfully improving the corrosion resistance by nearly fivefold and durability of the Mg alloy. By impregnating specimens into a Hank’s solution for 96 h the effects of calcium phosphate deposition were ascertained, with less corrosion products formed and no delamination detected (Al-Saadi et al., 2017). The impact of these findings shows that the incorporation of hBN into composites can improve the corrosion protection and antibacterial properties of biomedical devices, such as Mg-based ones, which have great biocompatibility and suitable mechanical properties for such purposes, but usually lack the necessary lifetime when in presence of human body fluids due to high corrosion rates (Riaz et al., 2019). The study of the approaches taken to tackle this problematic give insight into the dynamics occurring at the interface between these complex biological systems and the surface of the material, providing useful information on how to design better solutions. The action of these 2DM coatings in protecting the underlying substrate is twofold. On one hand, the reduced lattice size blocks the access of microbial communities to the substrate, thus hindering its corrosion during metabolic activity. On the other hand, the electrostatic properties of these materials have shown to successfully inhibit bacterial adhesion. The double effect here described can be exploited to prevent corrosion of implants.

### Graphene as Stem Cell Differentiation Promoter

**FIGURE 9:**
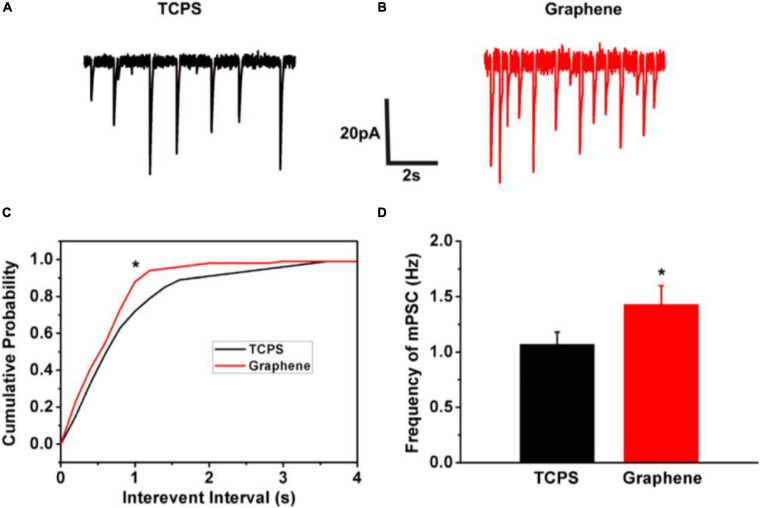
Graphene substrate increases miniature synaptic activity. **(A,B)** Representative miniature synaptic currents (mPSCs) are shown in both TCPS and graphene groups; **(C)** Cumulative probability plots of inter event interval and amplitude of mPSCs were presented in both TCPS and graphene groups. Histogram plots of mPSC frequency **(D)** and amplitude **(C)** were to identify the difference of selected index. Note the significant increase in the mPSC frequency (*p < 0.05) and no difference in the mPSC amplitude. Reprinted from Tang et al. (2013) Elsevier ltd.

Tissue engineering is a therapeutic multidisciplinary field that exploits medical, biological, and physical–chemical expertise with the purpose to obtain biomimetic tissues for restoring, recreating or improving the original physiological functions. The advent of nanomaterials represents an extraordinary research opportunity in this field, already featuring a large amount of publications and patents (Hubbell, 1995; Lutolf and Hubbell, 2005; Kohane and Langer, 2008). In the last decade, a large number of papers focusing on the capability of graphene to promote stem cell differentiation have been published. Park et al. investigated the effect of graphene on hNSCs, demonstrating that 10 h after cell feeding the majority of hNSCs adhered preferentially onto graphene, rather than onto glass (Figure 4). Interestingly, after a long-term differentiation process, the number of differentiated cells on graphene was significantly higher, with a large prevalence of neurons instead of astrocytes. Authors concluded that graphene promotes cell adhesion and drives stem cell differentiation toward a neuronal faith by creating a more suitable microenvironment for the stem cells (Park et al., 2011). Also, graphene nano-spheres seem capable to promote NCS differentiation. Tang et al. (2013) displayed that after 1 days in culture, cells properly migrated and started differentiating into mature neurons. The neuronal differentiation, evaluated in terms of neurites growth and extension, was followed until complete neuronal maturation by staining cells with β-tubulin and MAP-2 markers. In addition, through electrophysiological and live imaging techniques, authors reported that graphene was capable to enhance electrical network signaling (Figure 9) (Tang et al., 2013).

Kalbacova et al. (2010); Nayak et al. (2011), and Park et al. (2014) studied the possibility of promoting mesenchymal stromal cell differentiation using graphene. Kalbacova et al. (2010) showed that, 48 h after the plating, MSCs homogenously covered all the graphene substrate, while cells plated onto the control support (SiO_2_) formed separated islands. In addition, MSCs seeded onto graphene showed a spindle-shape morphology, allowing a higher proliferation with a possible differentiation toward osteoblast lineage. On the contrary, MSCs plated onto SiO_2_ presented a more polygonal cell shape, typical of non-differentiated cells. Author also tested the cell adherence and concluded that cells plated onto graphene showed a weaker and smaller contact with the support, corresponding to an active state of the cells (Kalbacova et al., 2010). Nayak et al. (2011) used CVD graphene, transferred onto four different supports: glass, silicon wafer, PET, and PDMS. Authors reported that mesenchymal cells maintained their peculiar spindle-shape across when plated onto Gr/glass and Gr/SiO_2_. Notably, cells plated onto PET and PDMS (graphene coated or non) showed rounded and irregular morphology, probably due to the poor adhesion. By adding in the maintaining medium a protein cocktail to favor osteogenic differentiation, the cells plated onto the graphene uncoated supports did not express protein markers of differentiation after 15 days of treatment; on the contrary MSCs plated onto graphene coated substrates remarkably differentiated into osteoblasts at a comparable rate to the differentiation with the bone morphogenic protein-2 (BMP-2) (Nayak et al., 2011). In addition, Park et al. (2014) demonstrated that, even in absence of chemical differentiation inducers, graphene presence was sufficient to induce MSC commitment toward the cardiomyogenic lineage. They attributed this phenomenon to the upregulation of extracellular matrix protein and cell signaling molecule expression (Park et al., 2014). Liu et al. (2016) studied the *in vivo* effects of a CVD-grown monolayer graphene-coated Ti disks implanted in the back subcutaneous area of nude mice. Authors incubated *in vitro* human adipose-derived stem cells (hASCs) and human bone marrow mesenchymal stem cells (hBMMSCs) on the top of their support before implanting them into the mice. No evident *in vivo* toxicity was reported; in addition, they demonstrated that CVD graphene favored cell adhesion, proliferation and differentiation toward an osteogenic faith, in accordance with Nayak et al. (2011). By epigenetic analysis, authors revealed that graphene had the ability to upregulate the osteogenesis associated genes by increasing tri-methylation of H3K4 (Nayak et al., 2011; Liu et al., 2016). Between 2017 and 2019, Xie et al. (2017) published two works evaluating the potential of graphene to promote osteogenic differentiation. In the first work, authors studied the possibility for graphene to promote dental pulp stem cell (DPSC) differentiation toward an odontogenic or osteogenic faith. They evaluated mineralization and differentiation of DPSC after 14 and 28 days and reported that cells grown onto graphene presented higher level of mineralization. In addition, odontoblastic genes resulted down-regulated and osteogenic genes and proteins were significantly up-regulated. Interestingly, cells plated onto control glass, but grown with medium obtain from graphene samples, showed the down regulation of odontoblastic genes, and associated with an increase in bone-related gene and protein. Authors concluded that graphene was not a material suitable for dental reconstruction, but appropriate for bone tissue engineering (Xie et al., 2017). Starting from the previous work, Xie et al. studied the capability of graphene to promote *in vivo* osteogenesis and the molecular mechanisms at the base of this process. Authors generated MSC-impregned graphene scaffolds and implanted them into immunocompromised (SCID) mice at 28 days of life. Without the addition of any osteogenic inducers, graphene scaffolds were able to promote osteogenic differentiation of MSC by increasing the expression of bone-related markers (RUNX2 and OPN) (Xie et al., 2019). Li K. et al. (2018) investigated the possibility to enhance the surface bioactivation of titanium alloys (Ti6-Al4-V) by a graphene coating to improve osteogenesis and osseointegration in an *in vivo* New Zealand white rabbit femoral condyle defect model. Animals were implanted with both a graphene-coated and a non-coating supports and their capability to enforce osteogenesis after 4, 12, and 24 weeks was evaluated. No evident cytotoxicity was reported in this work. Biomechanical testing, micro-computed tomography (Micro-CT) analyses and histological observations were performed. Authors demonstrated that microstructure parameters (i.e., bone volume/total volume fraction and mineral apposition rate) and the new bone formation were significantly enhanced in animals implanted with the graphene-coated Ti6-Al4-V (Li K. et al., 2018).

### Graphene as Excitable Cell Activity Enhancer

**FIGURE 10:**
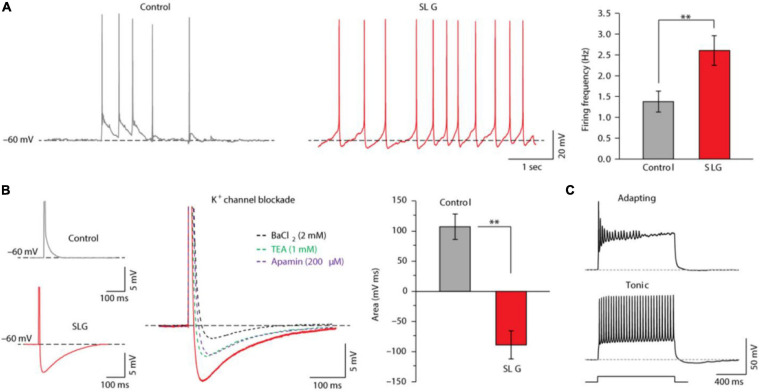
SLG triggers changes in single-cell intrinsic excitability. **(A)** Representative current-clamp recordings of hippocampal neurons in culture (10 DIV) in control and SLG. Control and SLG neurons displayed similar resting membrane potentials (–52 ± 10 mV in SLG; –50 ± 7 mV in control). When held at –60 mV, the cell’s spontaneous action potential firing was measured as summarized in the histograms (right). Note the significantly higher action potential frequency in SLG [(2.60 ± 0.36) Hz in SLG, *n* = 21; (1.37 ± 0.26) Hz in control, *n* = 19; *P* = 0.0054]. Significance: **P < 0.01. **(B)** Evoked single action potential in control (top) and SLG (bottom). Note the pronounced AHP in SLG neurons, which was partially abolished by each of the treatments shown: BaCl_2_, tetra-ethylammonium (TEA) or apamin (right, superimposed tracings). The histogram quantifies the area below the control and SLG post-AP voltage trajectories with respect to the resting membrane potential. The AHP in SLG neurons was significantly different from the ADP detected in control neurons [(–86.96 ± 23.60) mV ms in SLG, *n* = 25; (+107.12 ± 21.85) mV ms in control, *n* = 20; *P* = 0.0010]. Interestingly, the AHP was reduced (by 88%) by bath-applying Ba_2+_ (BaCl_2_, 2 mM; *n* = 3), which is known to block K_ir_ (inward-rectifier) and K_Ca_ (calcium-activated) membrane potassium channels (Alger and Nicoll, 1980**;** Jiang and MacKinnon, 2000**;** Alagem et al., 2001). The AHP was also reduced (by 58%) by bath-applying TEA (1 mM; *n* = 9), a non-selective blocker of the large majority of voltage-gated K^+^ membrane channels (K_v_) 47, including BK_Ca_ channels (Hille, 2001). Finally, apamin (200 μ M; *n* = 5), a specific inhibitor of SK_Ca_ membrane channels (Hille, 2001), also strongly affected the AHP (47% reduction). **(C)** Current-clamp recordings from neurons in control and SLG revealed different cell-discharge patterns, classified as adapting or tonic. Reprinted from Pampaloni et al. (2018) Springer Nature.

Due to its biocompatibility and electrical properties, graphene has been regarded as an advanced and viable interface for enhancing neuronal activity or improving the record of electrical cell signal. Li et al. demonstrated that CVD graphene is able to promote neurite sprouting and outgrowth (associated with a significant increase of GAP-43 protein expression), concluding that the graphene-coated substrate could have a prominent impact on the cell early developing stages (Li et al., 2011). In accordance with Li et al. (2011) and He et al. (2016) reported that graphene accelerated neuronal maturation, favoring microtubules formation and growth, and promoted dendritic spine density and maturation with a consequent enhanced synaptic transmission strength. They suggested that the acceleration in microtubules formation (known to play a pivotal role in neurite and axon specification, Kuijpers and Hoogenraad, 2011), coupled with graphene’s high electrical conductivity (as suggested by Tang et al., 2013), might be at the base of all the reported improvements. Kitko et al. (2018) displayed that neurons plated onto graphene showed an enhanced synaptic transmission, related to an increasing in the synaptic vesicle number, probability of release and recycling rate (Kitko et al., 2018). Pampaloni et al. (2018) testing the interaction between neurons and monolayer graphene, partially confirmed the capability of the graphene to enhance postsynaptic current (PSC) strength. In this work, however, the authors clearly demonstrated that the enhancement regarded only spontaneous synaptic transmission, without reporting any alteration in the synaptic bouton numbers and the miniature PSCs. In addition, they displayed that monolayer graphene could also increase neuronal firing activity, by regulation of the extracellular ion distribution around neurons. They demonstrated that neurons grown onto graphene showed a significant increase in potassium currents, responsible for the observed shift from adapting to tonically firing (Figure 10).

Through a theoretical approach, they speculated that monolayer graphene effects on neurons might be maximized when graphene is deposited in an electrically insulation support (i.e., glass), compared to a suspended condition. Authors concluded that the alteration in the spontaneous synaptic transmission could be justify by the different ratio between cells with adaptive/tonic phenotypes. Notably, authors did not exclude a potential contribution of astrocytes in the surface ion concentration alterations, since astrocytes are able to regulate the extracellular medium between neurons and the substrate (Pampaloni et al., 2018).

In a recent publication, Capasso et al. (2020) questioned the importance of conductivity as a crucial property to foster neuronal activity by investigating the electrical behavior of primary neurons plated onto graphene-based films. The authors prepared two films by CVD with different lattice structure and electrical conductivity. Neurons grown onto both substrates were able to form a highly structured and mature network. Interestingly, low-conductivity graphene seemed to improve the neuronal network architecture without significantly modifying intrinsic electrical activity, compared to pristine graphene. These results indicated that a high electrical conductivity *per se* is not sufficient to promote the electrical activity of neuronal networks, suggesting that other material features (e.g., surface chemistry, roughness, wettability, etc.) have to be taken into account in the design of future graphene-based implants (Capasso et al., 2020). Following this rationale, the same group demonstrated that monolayer graphene with higher hydrophilicity (water contact angle of 40.7° vs. 83.7° of the pristine graphene) improved cell-to-cell communication. The authors fabricated such highly hydrophilic coatings on polyethylene terephthalate using remote plasma hydrogenation, which did not affect graphene’s crystallinity allowing to preserve a residual electrical conductivity (∼3 kΩ/□). The formation of excitatory synaptic connections increased in hydrogenated graphene with respect to pristine graphene, leading to a doubled miniature excitatory postsynaptic current frequency. This study indicated that wettability might be the key to enable neuronal interfacing (Moschetta et al., 2020).

### Graphene for Biosensing

CVD graphene demonstrated excellent biocompatibility, as shown by its ability to drive neuronal growth and regeneration *in vivo* (Li et al., 2011). Due to its positive impact in cultured neurons, graphene revealed to be a durable and biocompatible material for intracortical probes due to the reduced proliferation of astrocytes and microglia. Healthier neuron network at the implant site provide a sustainable coupling between electrode and target neurons, allowing for chronic recording and to act as scaffolds for regenerative medicine. The acceptance of intracortical implant is crucial for neurorehabilitation applications, since reliable and long-lasting monitoring of single units in freely moving environment are required for replacing disable node of the neural network and restore sensory function or fine motor command (Bourrier et al., 2019a). Monitoring the activity of individual neurons requires the intimate contact of micro and nano electronic devices with cells over mesoscale networks. Gliosis is the immune response of cells and tissues that lead to the rejection of the penetrating intracortical probes, hindering the realization of neuro-rehabilitation projects. Graphene and other carbon-based nanomaterials have risen as good candidates due to good adhesion, neuron regeneration, and potential to provide highly sensitive devices such as graphene field effect transistors (GFET). Graphene’s mechanical properties also allow it to conform to soft tissues, further improving its biocompatibility and contributing to the acceptance of intracortical probes. However, monolayer-based devices could be teared off during implantation and manipulation and might degrade over time within the brain. Bourrier et al. have attempted to address this issue by supporting graphene with a biocompatible and degradable polymeric film (based on hyaluronic acid). The protective polymeric layer does not hinder device’s features and function, namely neuron and tissue response (Bourrier et al., 2019b). Veliev et al. have reported on the fabrication of GFETs on various substrates with high sensitivity and low noise level. Experiments carried out *in vitro* showed detection of spontaneous activity of hippocampal neurons (which were grown *in situ* on top of the graphene sensors). The hippocampal neuron cells exhibited healthy morphology and spontaneous electrical activity after 19–21 days in culture. The electrical measurements did not harm the cells. The performance of the GFETs degrades slightly with time (Veliev et al., 2017). The ultimate resolution of neural activity sensing consists of ion channel activity monitoring. Graphene has been used to monitor individual ion channels through field effect detection (Veliev et al., 2018). The advent of e-tattoos, or epidermal electronics, provided non-invasive and high-fidelity sensing, and graphene showed promise in this regard due to its mechanical and electronic properties (Kabiri Ameri et al., 2017). It has been also used in a self-healable tattoo that allows continuous monitoring of electrophysiological signals (e.g., skin temperature and hydration; glucose, urea, sodium, and calcium levels). Graphene has been used to create multifunction ultrathin tattoos. Enabled by graphene’s mechanical properties and biocompatibility, these tattoos provide high-fidelity sensing by conforming to the micro-roughness of the skin by matching its modulus. Skin/electrode contact interface is enlarged, lowering contact impedance and higher signal to noise ratios (Wang Q. et al., 2019).

Finally, DNA has been shown to bind directly to graphene without the need of a linker, making it highly interesting for biosensor development (Campos et al., 2018). Field-effect transistors (FET) with two-dimensional channels made of monolayer graphene have been developed to achieve label-free detection of DNA hybridization down to attomolar concentration, while offering at the same time the possibility of discriminating single nucleotide polymorphism (Campos et al., 2019). Additionally, graphene has been used to track DNA hybridization reaction with nanoscale resolution in real time by using nano-photonic effects (Adão et al., 2019).

## Conclusion

In this review, we reported on the interaction of graphene and hBN coatings with living cells, both bacterial and mammalian, focusing on the interaction mechanisms and accounting for current applications. Graphene and hBN are two-dimensional materials with analogous lattice structure and markedly different electrical properties. Although the literature on graphene for biomedical applications is more extensive, we chose to survey relevant research results on hBN to point out the current state of the art on both materials and compare the interaction mechanisms.

In the case of graphene coatings, an active antibacterial mechanism is observed which seems to be strongly dependent on the coating morphology, and consequently on the production method. On the one hand, liquid-phase produced graphene coatings are generally morphologically heterogeneous and present exposed flake edges, which have shown to interact with bacterial cells and lead to cellular impairment. This effect is dependent on orientation of the nanoflakes at the surface and effects certain bacterial species more than others, based on their size and shape, in accordance to the surface texture of the coatings. On the other hand, graphene films produced by chemical vapor deposition are usually planar, making in this case the basal plane at the root of the cellular interaction. Therefore, rather than edge dependent effects, CVD graphene coatings showed antibacterial effects due a high electrical conductivity, which would produce oxidative stress and a consequent depletion of ATP, culminating in bacteria cell death. The effectiveness of this charge transfer mechanism is dependent on the complexity of the cellular membrane.

With regards to mammalian cells, CVD graphene coatings produced revealed to be highly suitable for cell interfacing. Nonetheless, no universal consensus exists yet on the physio-chemical mechanism underlying the favorable interaction between cells and graphene, despite some authors attributed the excellent biocompatibility to the absence of ROSs formation or to the capability of graphene of directly interacting with specific molecules in the mammalian cell membrane (i.e., cholesterol). Overall, the absence of an evident *in vivo* cytotoxicity, in addition to the capability of boosting cell activity and stimuli responsivity, makes graphene an excellent candidate for the realization of cellular interfaces. In addition, graphene is found to promote cell adhesion and proliferation, and to drive stem cell differentiation toward both a neuronal and non-neuronal faith (depending on the stem cell subtype), by creating a suitable microenvironment for the stem cells. When interfacing neuronal stem cells, a graphene coating is able *per se* to boost neurite sprouting and maturation and, when interaction with mesenchymal stem cells, drive their maturation specifically toward osteoblast or cardiomyogenic lineage in both *in vitro* and *in vivo* systems. Besides its ability to favor stem cell maturation, when interfacing excitable cells, monolayer graphene films revealed also to enhance synaptic transmission by increasing spontaneous intracellular Ca^2+^ spikes and neuronal firing by altering extracellular K^+^ concentration. Traditionally, authors attributed these phenomena to the high electrical conductivity graphene. However, this feature alone is not enough to promote the electrical activity of neuronal networks, and hence other characteristics of the coating (e.g., thickness and roughness) must be considered when designing graphene-based interfaces and implants. As a final and crucial remark, the *in vivo* biocompatibility of graphene is still debated. Few works reported in the last decade alert about the possible *in vivo* toxicity of graphene and its derivatives. A peculiar aspect that troubles the scientific community regards the possibility of graphene to permanently deposits in the organs (lung, spleen and liver), causing chronic inflammatory processes. Nonetheless, the long-term persistence of graphene in the organisms has not been clearly demonstrated and in most of the cases the toxicity reduces over time; in addition, phenomena of graphene clearance through feces has been reported. On the other hand, the possibility to use 2D graphene as an *in vivo* therapeutical tool is still an open question. Graphene-coated prosthesis has been successfully employed to favor bone tissue regeneration; in addition, cancer cell aggregations seem permeable to graphene sheets and capable to retain them. This interesting phenomenon leads researcher to investigate the possibility to use graphene in the antineoplastic therapies.

As a partial conclusion about the mechanisms of interaction with the two kinds of cells, we underline that although a graphene coating in certain conditions can induce bacterial cell death, the mammalian cells seem to be generally unharmed by it. Based on these interactions, applications for graphene have surfaced. CVD graphene coatings have shown to be able to hinder bacterial adherence and coating due to electrostatic interactions at the surface. Additionally, its lattice size allows it to be impermeable to Cu and Ni ions, blocking the bacteria at the surface to metabolize the substrate. CVD graphene has also been used for sensing applications in neuroscience and medicine in the form of sensors, wearable devices and scaffolding due to its softness, electrical conductivity, and biocompatibility.

Due to the limited availability of literature regarding hBN coatings and their interaction with bacteria and cells, we have reviewed not only cases where this material was deposited on a substrate, but also those investigating its behavior in liquid solutions and in nanocomposites, in order to provide a more comprehensive overview. In the case of 2D hBN coatings, studies conducted experimentally with LPE samples and via simulations showed antibacterial effects related to the exposed flake edges (as shown for graphene), which promoted cellular membrane disruption and temperature-dependent lipid extraction. However, studies conducted in atomically smooth hBN coatings (e.g., CVD) have revealed no antibacterial activity of the basal plane, further highlighting the role of a sufficiently high electrical conductivity in bacterial death. Electrostatic interactions seemed to contribute toward an inhibition of bacterial adhesion. Despite their markedly different electrical conductivity, both graphene and hBN coatings have shown to be effective against biocorrosion of metal substrates by reducing the number of adhered bacteria, with the reduced cellular adhesion being consequence of the atomic smoothness of the coatings. In both cases, the small lattice size in graphene and hBN allows them to act as impermeable barriers, blocking the migration of aggressive metabolites and avoiding biocorrosion of the underlying metallic substrate.

With regards to mammalian cells, hBN has shown to be cytotoxic in liquid solution above certain concentrations and in a shape-dependent manner. Cytotoxicity studies conducted with hBN in liquid solution for different nanoflake sizes show that when the size is small enough it becomes possible for mammalian cells to internalize them and cause an increase in ROS production. Overall, 2D hBN materials have shown promise in bone tissue scaffolding, wound healing, and treatment of neurodegenerative diseases such as Parkinson’s. For further understanding the potential of this kind of materials, the cytotoxicity will need to be ascertained to address some of the inconsistencies present in literature.

In conclusion, the current scenario suggests that, notwithstanding the substantial amount of studies in the field, further investigation is still needed before two-dimensional materials such as graphene and hBN can fulfill their potential in advanced biomedical applications. This is particularly true in the case of hBN, where most interactions with living matter still needs to be understood and detailed.

## Author Contributions

JS and MM: data acquisition and writing original draft. JR: data revision and support on writing. PA: data interpretation and evaluation. AC: conceptualization, coordination, writing, and funding acquisition. All authors: final writing.

## Conflict of Interest

The authors declare that the research was conducted in the absence of any commercial or financial relationships that could be construed as a potential conflict of interest.
